# Association of CSF AD biomakers and lifestyle factors with neuropsychiatric symptoms among non‐demented older persons

**DOI:** 10.1002/alz.088304

**Published:** 2025-01-03

**Authors:** Anja Soldan, Valerie Truesdell, Corinne Pettigrew, Abhay Moghekar, Vadim Zipunnikov, Adam P. Spira, Marilyn S. Albert, BIOCARD RESEARCH TEAM

**Affiliations:** ^1^ Johns Hopkins University School of Medicine, Baltimore, MD USA; ^2^ Johns Hopkins Bloomberg School of Public Health, Baltimore, MD USA; ^3^ Johns Hopkins University, Baltimore, MD USA

## Abstract

**Background:**

Higher level of cognitive reserve (CR), measured using proxies such as years of education or literacy, is associated with reduced risk of Mild Cognitive Impairment (MCI) and dementia. Little is known about how CR and other lifestyle factors impact non‐cognitive outcomes, including depression and other neuropsychiatric symptoms (e.g., agitation, delusions, anxiety, etc.). This study examined whether factors that influence cognition among non‐demented older adults (i.e., CR, demographics, vascular risk, sleep quality, physical activity, ApoE4 genetic status) are also associated with depression and other neuropsychiatric symptoms, and whether these associations vary by AD pathology levels.

**Method:**

Depression and other neuropsychiatric symptoms were assessed using the Geriatric Depression Scale (GDS) and Neuropsychiatric Inventory (NPI), among 181 non‐demented BIOCARD Study participants (mean age = 72.6y, including 152 cognitively unimpaired and 29 with MCI). Total volume of physical activity and sleep efficiency were measured using actigraphy. Level of CR was based on a composite of years of education, vocabulary, and reading ability. A subset of participants (n = 125) had cerebrospinal fluid measures of amyloid and tau (Fujirebio Lumipulse G1200 assays), with AD pathology levels summarized as the ratio of p‐tau181/(AB42/AB40). Linear regressions evaluated the cross‐sectional relationship between the risk and protective factors with total GDS and NPI scores, and interactions with AD‐pathology levels.

**Results:**

For depression, greater total physical activity (p<0.004) and greater sleep efficiency (p = 0.006) were associated with lower GDS scores, independent of CR, age, sex, vascular risk, and ApoE4 status, which were not associated with GDS scores. The strength of this association was reduced among participants with higher AD‐pathology levels (p<0.05 for interactions of ptau_181_/(Ab_42_/Ab_40_ with physical activity and sleep efficiency. For neuropsychiatric symptoms, higher CR scores were associated with lower NPI scores and this association was independent of p‐tau181/(AB42/AB40) levels, but greater among those with poor sleep efficiency and among men.

**Conclusion:**

These results suggest that level of cognitive reserve, sleep quality, and physical activity levels are associated with depression and other neuropsychiatric symptoms among non‐demented older adults. Moreover, these associations differentially depend on AD‐biomarker levels, suggesting that individualized approaches to the treatment of neuropsychiatric symptoms among older adults are likely most successful.

